# The future burden of obesity in Canada: a modelling study

**DOI:** 10.17269/s41997-019-00251-y

**Published:** 2019-08-19

**Authors:** Meghan O’Neill, Kathy Kornas, Laura Rosella

**Affiliations:** 1 Dalla Lana School of Public Health, University of Toronto, 155 College Street, Health Sciences Building 6th Floor, Suite 600, Toronto, ON M5T 3M7 Canada; 2 Institute for Clinical Evaluative Sciences, Room 424, 155 College Street, Toronto, ON Canada; 3 Public Health Ontario, 480 University Avenue, Suite 300, Toronto, ON M5G 1V2 Canada

**Keywords:** Population health, Obesity, Chronic disease, Community health planning, Forecasting, Santé de la population, Obésité, Maladie chronique, Planification de la santé communautaire, Prévision

## Abstract

**Objectives:**

We applied the validated Obesity Population Risk Tool (OPoRT) to estimate the future burden of obesity in Canada using baseline risk factors attained through routinely collected survey data.

**Methods:**

OPoRT was developed using logistic regression with sex-specific generalized estimating equations to predict the 10-year prevalence of obesity (outcome BMI ≥ 30.0) among adults 18 and older. The algorithm includes 17 predictive factors, including socio-demographic and health behavioural characteristics. OPoRT demonstrated excellent discrimination (*C*-statistic ≥ 0.89) and achieved calibration. We applied OPoRT to Canadian Community Health Survey (2013/14) data to predict the future prevalence of obesity in Canada for a variety of population subgroups.

**Results:**

The predicted burden of obesity grew from 261 cases per 1000 in 2013/14 to 326 cases per 1000 in 2023/24 corresponding to a total of 8.54 million individuals with obesity. The burden is expected to be higher among males (347 cases per 1000) than females (305 cases per 1000). Individuals aged 35–49 had the highest predicted burden of obesity (374 cases per 1000) and the largest number of predicted cases (2.42 million), while individuals in the ≥ 65 age group had the lowest predicted burden (236 cases per 1000). The number of individuals with obesity per 1000 is highest among those severely food insecure (452 cases per 1000), compared with food secure individuals (324 cases per 1000).

**Conclusions:**

OPoRT can be used to estimate the future population burden of obesity, to identify priority subgroups at an elevated risk. Burden estimates should be reflected in approaches to curb the future burden of obesity.

**Electronic supplementary material:**

The online version of this article (10.17269/s41997-019-00251-y) contains supplementary material, which is available to authorized users.

## Résumé

**Objectifs:**

Nous avons appliqué l’OPoRT, un outil validé d’estimation de l’obésité dans la population, pour estimer le fardeau futur de l’obésité au Canada à l’aide de facteurs de risque de référence obtenus au moyen de données d’enquête collectées systématiquement.

**Méthode:**

L’outil OPoRT a été élaboré par régression logistique avec des équations d’estimation généralisées sexospécifiques pour prédire la prévalence de l’obésité dans 10 ans (IMC résultant ≥ 30,0) chez les adultes de 18 ans et plus. L’algorithme comporte 17 facteurs prédictifs, dont des caractéristiques sociodémographiques et des caractéristiques comportementales liées à la santé. L’outil OPoRT a présenté une excellente capacité de discrimination (indice de concordance ≥ 0,89) et a permis d’effectuer un étalonnage. Nous l’avons appliqué aux données de l’Enquête sur la santé dans les collectivités canadiennes (2013-2014) pour prédire la prévalence future de l’obésité au Canada dans divers segments démographiques.

**Résultats:**

Le fardeau prévu de l’obésité passerait de 261 cas p. 1 000 en 2013-2014 à 326 cas p. 1 000 en 2023-2024, ce qui correspond à 8,54 millions de personnes obèses en tout. Il devrait être plus élevé chez les hommes (347 cas p. 1 000) que chez les femmes (305 cas p. 1 000). Les personnes de 35 à 49 ans présenteraient le fardeau d’obésité prévu le plus élevé (374 cas p. 1 000) et le plus grand nombre de cas prédits (2,42 millions), et les personnes de ≥ 65 ans, le plus faible fardeau d’obésité prévu (236 cas p. 1 000). Le nombre de personnes obèses p. 1 000 serait le plus élevé chez les personnes en situation d’insécurité alimentaire grave (452 cas p. 1 000) comparativement aux personnes à l’abri de l’insécurité alimentaire (324 cas p. 1 000).

**Conclusions:**

L’outil OPoRT peut servir à estimer le fardeau futur de l’obésité dans la population et à définir les sous-groupes prioritaires courant un risque élevé. Les estimations de ce fardeau devraient se traduire par des démarches pour résorber le fardeau futur de l’obésité.

## Introduction

Obesity is a key contributor to the burden of disease in both developed and developing nations, making the management and prevention of obesity in Canada a top public health priority (Janssen 2013). An established body of literature shows that obesity is associated with an increased risk of many chronic conditions, including hypertension, type 2 diabetes, sleep apnea, and certain types of cancer (Guh et al. 2009). In addition, obesity is shown to affect quality of life, education, and income potential, and increase the risk of premature death (Puhl and Heuer 2009).

Forecasting the future burden of obesity among population subgroups is one way to help inform more effective obesity prevention strategies. This can be accomplished through the application of population risk algorithms that are designed to describe the distribution of risk, predict the number of people who will develop a disease or condition, and estimate the benefit of health interventions (Manuel et al. 2012; Califf and Harrell 2018). The Obesity Population Risk Tool (OPoRT) is one such validated tool that was developed in Canada as a means to estimate population trajectories of obesity based on the distribution of risk factors that are routinely collected in population surveys (Lebenbaum et al. 2018).

There are a number of existing prediction models to identify individuals at an increased risk for several obesity-related diseases that include cardiovascular disease (Conroy et al. 2003), type 2 diabetes (Rosella et al. 2011), and cancer (Usher-Smith et al. 2018; Winkler Wille et al. 2015). However, there are limited tools in practice that have the capacity to assess future burden of chronic disease risk factors. Population-based risk prediction algorithms, such as OPoRT, are novel in their wide applicability to be run on population health surveys, while incorporating individual-level risk factor data.

To our knowledge, this is the first application of a risk prediction model for obesity at the Canada-wide population level. The prevention of obesity is particularly important given the large disease burden in combination with the limited long-term effectiveness of most public health interventions (Hafekost et al. 2013). The objective of this study was to apply the OPoRT to the 2013/14 Canadian Community Health Survey (CCHS) to estimate the future burden of obesity, identify subgroups at an elevated risk, and demonstrate the use of a tool that can be used to inform obesity prevention.

## Methods

### Data sources and study population

For this study, we applied the OPoRT to risk factor information from the Canada-wide 2013/14 CCHS. The CCHS is a nationally representative household survey of Canadians conducted by Statistics Canada which collects information on health status, determinants of health, and health care utilization. It covers 98% of the Canadian population aged 12 years and older; exceptions include people living on First Nations Reserves and Crown Lands, institutionalized residents, full-time members of the Canadian Forces, and people who reside in certain remote areas. All responses to questions were self-reported. Detailed descriptions of the CCHS survey methodology are documented elsewhere (Statistics Canada 2018).

A total of 121,486 individuals responded to the CCHS. We restricted our analyses to individuals ≥ 18 years of age at baseline (*n* = 111,772). We further excluded pregnant women from the sample due to the limited accuracy of body weight measurements (*n* = 110,825), and individuals with missing BMI, resulting in a final sample size of 105,297.

### Statistical analysis

To estimate the predicted burden and number of total cases of obesity over the next 10 years, we used the OPoRT. This is a sex-specific predictive algorithm developed to calculate future population burden of obesity (body mass index ≥ 30.0 kg/m^2^) among adults 18 years and older. The logistic regression model was previously developed and validated using data from the National Population Health Survey using sex-specific generalized estimating equations. The model was validated using data from 10 years of follow-up (2005–2006, NPHS Cycle 7) and 0.632+ Bootstrap method to assess the potential optimism of the model. The model achieved excellent discrimination (*C*-statistic = 0.890 for males and *C* = 0.918 for females) and was well calibrated during the validation. Overall Brier score was 0.115 for males and 0.090 for females. To ensure the model was representative of the Canadian population, survey weights were incorporated in the analysis, which also take into account non-response rates at baseline and follow-up. Full details on the model specification and validation have been previously published (Lebenbaum et al. 2018).

Predictive variables used in the OPoRT algorithm include baseline BMI, age, obesity, time, smoking status, living arrangements, any post-secondary education (males), non-drinking status (males), physical inactivity (females) and ethnicity (females). The sex-specific OPoRT functions for males and females can be found in Online Resource 1. No covariate was missing in more than 2.41% of cases and the total proportion of missing in our dataset was 5.25%. For individuals missing covariate information that is required for the probabilities calculation (i.e., missing information on at least one variable required for the calculation), they were assigned the mean predictive probability from the overall cohort. This approach was chosen because it would not change the overall predicted risk and would allow for the number of cases to reflect the entire population without excluding those with missing values, which is important for estimating obesity burden. In addition, we conducted sensitivity analysis where each missing variable in the predictive model was assigned the most frequent category, as recommended by Harrell (Harrell 2001), to examine the impact of missing data. Calculation of BMI (kg/m^2^) was based on the World Health Organization (WHO) cut-offs (World Health Organization 2000). BMI correction equations were applied to provide more accurate estimates of an individual’s true weight status (Shields et al. 2011). Food security was an optional CCHS module that was only asked in certain provinces across Canada. Information on food security was not collected in Newfoundland and Labrador, Manitoba, British Columbia, and Yukon and as a result, the food security variable is only reflective of nine provinces and territories. All other variables examined were present in all provinces and territories.

Descriptive statistics were calculated for socio-demographic and health behaviours at baseline (i.e., CCHS interview year) by obesity status and the overall cohort population. OPoRT was used to estimate the 10-year predicted obesity burden by important population subgroups, including sex, age group (< 35, 35–49, 50–64, 65+), ethnicity (white, visible minority), immigration status (Canadian born, immigrant < 10 years, immigrant ≥ 10 years), household food security (food secure, moderately food insecure, severely food insecure), smoking status (heavy smoker, light smoker, former heavy smoker, former light smoker, and non-smoker), physical activity (physically active, physically inactive), alcohol consumption (never drinker, light drinker, moderate drinker, heavy drinker), number of health risk behaviours (including former or heavy and light or current smoker, moderate or heavy alcohol consumption, and physical inactivity), self-perceived general health (excellent, very good, good, fair, poor), self-perceived life stress (not at all stressful, not very stressful, a bit stressful, quite a bit stressful, extremely stressful) and number of chronic conditions (self-reported as having any of the following: asthma, arthritis, back problems, migraines, chronic obstructive pulmonary disease, diabetes, hypertension, heart disease, cancer, intestinal ulcers, stroke, urinary incontinence, bowel disease, mood disorder, or anxiety). The number of health risk behaviours was calculated by combining self-reported smoking (current or former heavy or light smoker), alcohol consumption (moderate or heavy), and physical inactivity (< 1.5 METs/day). The subgroups used in the analyses were chosen because they were considered in our original development and validation paper as established risk factors for obesity (Lebenbaum et al. 2018). A list of CCHS questions used to define descriptive stratification variables can be found in Online Resource 2.

OPoRT was used to estimate the 10-year predicted obesity burden by important population subgroups. Burden of obesity was calculated by multiplying individual probabilities estimated by OPoRT (ranging from 0 to 1) by 1000. Statistics Canada sample weights were applied to each individual probability to generate the number of future cases of obesity that is reflective of the Canadian population. In addition, geographic trends in the predicted burden of obesity were examined at a provincial level. Bootstrap sampling weights, provided by Statistics Canada, were applied using balanced repeated replication (BRR) to all analyses to adjust for the complex survey design of the CCHS and to produce estimates reflective of the Canadian population (Statistics Canada 2018). Weighted 95% confidence limits were calculated for descriptive baseline characteristics and for the predicted population subgroup probabilities. All statistical analyses were performed using SAS version 9.4 (SAS Institute Inc.; Cary, NC).

## Results

Among the CCHS 2013/14 survey respondents, *n* = 105,297 individuals (*n* = 57,827 females, *n* = 47,470 males) were eligible for inclusion and had an OPoRT burden generated. Table 1 provides the weighted descriptive frequencies to characterize the study population. The burden of obesity in Canada in 2013/14 was 261 cases per 1000 corresponding to a total of 6.84 million individuals with obesity. At interview, more males (14.0%) than females (12.1%) were classified as having obesity. Those with obesity were more likely to be of white ethnicity, Canadian born, between the ages of 50 and 64, have less than post-secondary education, and were self-reportedly physically inactive. Individuals who were non-smokers and light smokers were more likely to not be classified with obesity at baseline, compared with those who were heavy smokers, former (heavy) smokers and former (light) smokers. We conducted a sensitivity analysis using an alternative approach to examine the impact of missing data. The results of the sensitivity analysis and the results presented did not differ substantively (see Online Resource 3).

**Table 1:** Weighted distribution of baseline characteristics across CCHS 2013/14 cohort

	Overall (*N* = 105,297)		Obese at baseline (*N* = 30,932; 26.1%)		Not obese at baseline (*N* = 74,365; 73.9%)	
Weighted *N*	26,199,082		6,843,917		19,355,165	
Risk factor	%	95% CI	%	95% CI	%	95% CI
Sex (male)	50.2%	(50.1, 50.4)	54.0%	(52.6, 54.5)	49.0%	(48.7, 49.4)
Sex (female)	49.8%	(49.6, 49.9)	46.5%	(45.5, 47.4)	51.0%	(50.6, 51.3)
Age group (years)						
< 35	28.3%	(28.0, 29.7)	20.4%	(19.6, 21.3)	31.1%	(30.7, 31.6)
35–49	25.9%	(25.3, 26.4)	27.6%	(26.5, 28.7)	25.2%	(24.7, 25.8)
50–64	27.6%	(27.2, 28.1)	33.1%	(32.1, 34.2)	25.7%	(25.2, 26.2)
65+	18.2%	(18.1, 18.3)	18.8%	(18.2, 19.4)	18.0%	(17.7, 18.2)
Ethnicity						
White	77.1%	(76.4, 77.8)	83.0%	(82.0, 84.1)	75.0%	(74.2, 75.8)
Visible minority	22.9%	(22.2, 23.6)	17.0%	(15.9, 18.0)	25.0%	(24.2, 25.8)
Immigration status						
Canadian born	72.3%	(71.5, 73.0)	78.5%	(77.4, 79.6)	70.0%	(69.2, 70.9)
Immigrant	24.5%	(23.7, 25.2)	18.6%	(17.5, 19.6)	26.5%	(25.7, 27.4)
Household education						
Less than secondary	12.4%	(12.1, 12.8)	14.8%	(14.2,15.5)	11.6%	(11.2, 12.0)
Secondary school graduation	20.3%	(19.9, 20.8)	21.8%	(20.9, 22.7)	19.8%	(19.3, 20.3)
Some post-secondary	5.6%	(5.4, 5.9)	5.3%	(4.8, 5.8)	5.7%	(5.5, 6.0)
Post-secondary graduation	60.1%	(59.6, 60.6)	56.6%	(55.5, 57.6)	61.3%	(60.7, 62.0)
Equivalized household income						
Lowest	19.0%	(18.5, 19.6)	18.6%	(17.7, 19.5)	19.2%	(18.6, 19.8)
Low-middle	19.8%	(19.4, 20.3)	20.0%	(19.2, 20.7)	19.8%	(19.3, 20.3)
Middle	20.0%	(19.7, 20.5)	20.7%	(19.8, 21.6)	19.9%	(19.4, 20.4)
High-middle	20.1%	(19.7, 20.6)	20.1%	(19.2, 21.1)	20.1%	(19.6, 20.7)
Highest	20.8%	(20.4, 21.3)	20.6%	(19.8, 21.4)	21.0%	(20.4, 21.5)
Smoking status						
Heavy smoker (1+ packs/day)	2.7%	(2.6, 2.9)	3.4%	(3.0, 3.8)	2.5%	(2.3, 2.7)
Light smoker (< 1 pack/day)	16.3%	(15.9, 16.7)	15.0%	(14.3, 15.8)	16.8%	(16.2, 17.3)
Former (heavy) smoker	6.1%	(5.9, 6.3)	9.8%	(9.2, 10.3)	4.8%	(4.5, 5.0)
Former (light) smoker	17.5%	(17.1, 18.0)	19.4%	(18.6, 20.2)	16.9%	(16.3, 17.4)
Non-smoker	53.6%	(53.0, 54.2)	48.7%	(47.6, 49.9)	55.4%	(54.7, 56.0)
Physical activity						
Physically active (≥ 1.5 METs/day)	53.5%	(52.9, 54.1)	44.4%	(43.4,45.4)	56.7%	(56.0, 57.4)
Physically inactive (< 1.5 METs/day)	46.5%	(45.9, 47.1)	55.6%	(54.6,56.6)	43.3%	(42.6, 44.0)
Alcohol consumption						
Never drinker	19.2%	(18.8, 19.7)	20.0%	(19.17, 20.8)	19.0%	(18.4, 19.5)
Light drinker	16.3%	(15.9, 16.7)	19.5%	(18.6, 20.3)	15.2%	(14.7, 15.7)
Moderate drinker	24.2%	(23.8, 24.7)	21.9%	(21.0, 22.7)	25.1%	(24.6, 25.6)
Heavy drinker	40.2%	(39.7, 40.8)	38.7%	(37.6, 39.7)	40.8%	(40.1, 41.4)

Weighted using bootstrap weights as described by Statistics Canada. Column percentages do not total 100% where missing values are not reported

Table 2 presents the predicted burden of obesity (cases per 1000) and the total predicted number of cases of obesity according to different population subgroups weighted to reflect the Canadian population. Using OPoRT, we estimated that the 10-year burden of obesity in Canada will be 326 cases per 1000, corresponding to a total of 8.54 million obese individuals by 2023/24, an increase of 1.70 million cases from 2013/14.

**Table 2:** Ten-year predicted obesity burden among adult Canadians by socio-economic, behavioural and health status characteristics (2013/14–2023/24)

Characteristic	Predicted burden of obesity by population subgroup (cases per 1000)	Total predicted number of cases with obesity (thousands)
Total predicted number of obese cases	326 (322,330)	8540
Socio-economics		
Sex		
Male	347 (340, 352)	4550
Female	305 (301, 311)	3990
Age group		
< 35	329 (321, 336)	2440
35–49	374 (364, 381)	2520
50–64	337 (329, 343)	2435
65+	236 (231, 245)	1147
Ethnicity		
White	338 (334, 342)	6615
Visible minority	286 (278, 297)	1669
Immigrant status		
Canadian born	349 (344, 353)	6602
Immigrant	262 (252, 271)	1679
Food security*		
Food secure	324 (319, 328)	6204
Moderately food insecure	405 (384, 425)	402
Severely food insecure	452 (414, 488)	215
Behavioural		
Smoking status		
Heavy smoker (1+ packs/day)	396 (370, 415)	282
Light smoker (< 1 pack/day)	343 (333, 353)	1465
Former (heavy) smoker	419 (402, 426)	659
Former (light) smoker	336 (328, 345)	1543
Non-smoker	304 (299, 310)	4284
Physical activity^†^		
Physically active (≥ 1.5 METs/day)	294 (289, 298)	4108
Physically inactive (< 1.5 METs/day)	364 (358, 370)	4431
Alcohol consumption		
Never drinker	321 (312, 329)	1580
Light drinker	364 (354, 374)	1521
Moderate drinker	275 (268, 281)	1704
Heavy drinker	345 (339, 351)	3556
Number of health risk behaviours (current/former, heavy/light smoking, alcohol consumption (moderate or heavy) and physical inactivity)		
0	281 (269, 291)	784
1	305 (299, 311)	3177
2	346 (340, 352)	3284
3	372 (361, 383)	1298
Health status		
BMI category		
Overweight (BMI 25.0–29.9)	288 (284, 291)	2676
Self-perceived general health		
Excellent	225 (222, 236)	1242
Very good	308 (303, 315)	3106
Good	388 (378, 392)	2989
Fair	409 (392, 417)	899
Poor	415 (382, 436)	301
Self-perceived life stress		
Not at all stressful	295 (285, 305)	874
Not very stressful	307 (300, 314)	1835
A bit stressful	328 (322, 333)	3642
Quite a bit stressful	352 (343, 361)	1817
Extremely stressful	388 (365, 409)	351
Number of chronic conditions^‡^		
0	289 (282, 294)	3268
1	325 (317, 332)	2174
2	349 (339, 357)	1337
3	386 (374, 398)	777
4	416 (399, 432)	419
5	446 (423, 469)	216
6 or more	498 (470, 526)	207

*Excluding Newfoundland and Labrador, Manitoba, British Columbia, and Yukon

^†^METs are metabolic equivalent of task (kcal/kg/day). For example, the “inactive” physical activity is equal to walking for exercise less than 30 min per day (3 METS/h)

^‡^Chronic conditions include self-reported asthma, arthritis, back problems, migraine headaches, chronic obstructive pulmonary disease, diabetes, hypertension, heart disease, cancer, intestinal ulcers, stroke, urinary incontinence, bowel disorder, mood disorder, and anxiety disorders

The burden is expected to be higher among males than females (347 cases per 1000 compared with 305 cases per 1000). The age group with the highest predicted burden of obesity is expected to be ages 35–49 (374 cases per 1000), which also represents the age group with the largest total predicted number of cases (*n* = 2.52 million). Predicted burden of obesity according to ethnicity and immigrant status show that respondents who reported being white and Canadian born are expected to experience a greater burden of obesity than visible minorities and immigrants (338 cases per 1000 compared with 286 cases per 1000; 349 cases per 1000 compared with 262 cases per 1000, respectively).

Among the respondents of the included regions, people who were food insecure were predicted to have approximately 40% more burden of obesity than respondents who were food secure (324 cases per 1000 compared with 452 cases per 1000). Respondents who reported being former heavy smokers were predicted to have the greatest burden of obesity (419 cases per 1000) compared with non-smokers (304 cases per 1000), amounting to an over 25% relative difference between the two categories. Examining the future burden of obesity by physical activity shows that those who reported being physically inactive have an increased burden of obesity compared with those who were physically active (364 cases per 1000 compared with 294 cases per 1000).

Using OPoRT, we estimated that those who reported being light drinkers will have a higher predicted burden of obesity (364 cases per 1000) compared with never drinkers (321 cases per 1000). However, the greatest total number of persons with obesity will occur in the heavy drinker category (*n* = 3.56 million) with a burden of 345 cases per 1000. In combining responses from three health risk behaviours (smoking, alcohol consumption, and physical inactivity), we observe a dose-response increase in burden of obesity as the number of health risk behaviours increased from zero (281 cases per 1000) to three (372 cases per 1000).

Examining weight status at baseline shows that just over 30% of respondents who were classified as overweight are predicted to transition into the obese weight category (*n* = 2.68 million). Trends among health status indicators show a linear increase in burden of obesity as indicators of overall health status decline. For example, respondents who rated their self-perceived general health as poor had nearly twice the burden of obesity compared with respondents who rated their self-perceived general health as excellent (415 cases per 1000 compared with 225 cases per 1000). This trend is also observed for self-perceived life stress, although the jump between extremely stressful and not at all stressful is a smaller amount (388 cases per 1000 compared with 295 cases per 1000). As expected, as the number of chronic conditions increases, the burden of obesity increased from 289 cases per 1000 among the category of zero chronic conditions to 498 cases per 1000 in the 6 or more chronic conditions category. The largest total burden of obesity is expected to occur among those with zero chronic conditions (*n* = 3.27 million).

**Fig. 1:**
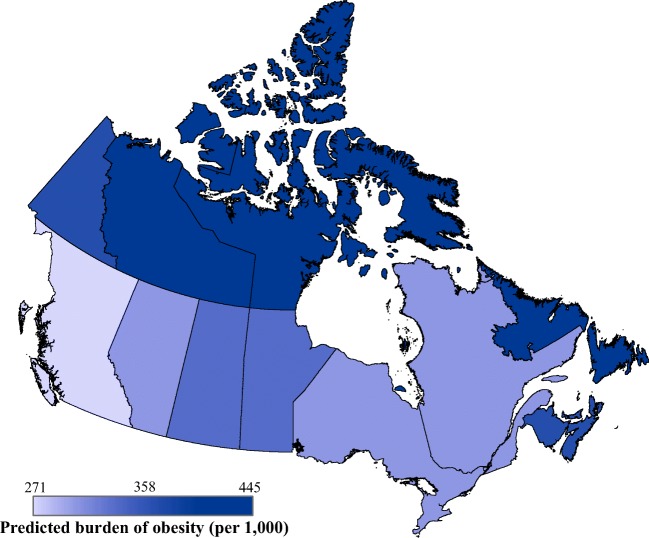
Predicted burden of obesity among adults in Canada, by province and territory (2013/14–2023/24)

The predicted number of persons with obesity (per 1000) by province in Canada is presented in Fig. 1, detailing associations between geographic location and obesity burden. The highest burden of obesity is predicted to occur in the Northwest Territories (445 cases per 1000), Nunavut (444 cases per 1000), Nova Scotia and New Brunswick (401 cases per 1000 for both provinces). The province with the lowest burden of obesity is predicted to be British Columbia (271 cases per 1000), followed by Quebec (315 cases per 1000), and Ontario (322 cases per 1000).

**Fig. 2:**
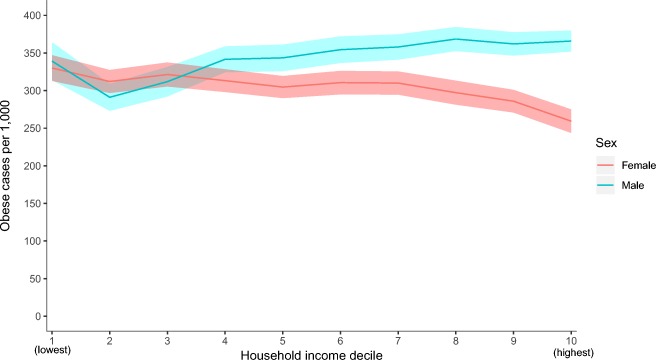
Predicted burden of obesity among Canadian adults by sex and household income decile (2013/14–2023/24)

The burden of obesity across household income deciles is depicted in Fig. 2. Predictions from OPoRT show a negative association between income and obesity for women, where as income increases, the burden of obesity decreases. This trend is reversed in men where a positive association is observed such that as income increases, burden of obesity also increases.

Differences between the baseline number of people with obesity compared with the 10-year risk is presented in Table 3. Notably, the < 35 age group is predicted to see the largest increase in persons with obesity (1.04 million) with the largest reduction in persons with obesity among those 65+ (− 141 thousand). Those who are self-reportedly food secure are predicted to contribute the largest number of new cases of obesity over the 10-year period (1.22 million), compared with those who are severely food insecure (39.9 thousand). Individuals with 1 health risk behaviour are expected to have the largest increase in cases with obesity (709 thousand new cases), followed by those with 2 health risk behaviours (610 thousand new cases). Last, those who did not report any chronic conditions are predicted to see the largest increase in obesity with 1.09 million new cases over the 10-year period.

**Table 3:** Ten-year predicted change in burden of obesity among adult Canadians by socio-economic, behavioural and health status characteristics (2013/14–2023/24)

Characteristic	Total baseline number of cases with obesity (thousands)	Total predicted number of cases with obesity (thousands)	Incident number of cases with obesity (thousands)
Total predicted number of obese cases	6844	8543	1699
Socio-economics			
Sex			
Male	3663	4553	891
Female	3181	3990	809
Age group			
< 35	1399	2440	1041
35–49	1889	2521	632
50–64	2269	2435	166
65+	1288	1147	− 141
Ethnicity			
White	5517	6615	1099
Visible minority	1129	1669	540
Immigrant status			
Canadian born	5374	6602	1228
Immigrant	1271	1679	408
Food security*			
Food secure	4981	6204	1223
Moderately food insecure	320	402	82.1
Severely food insecure	175	215	39.9
Behavioural			
Smoking status			
Heavy smoker (1+ packs/day)	231	282	51.2
Light smoker (< 1 pack/day)	1030	1465	435
Former (heavy) smoker	668	659	− 8.89
Former (light) smoker	1328	1543	214
Non-smoker	3336	4284	948
Physical activity^†^			
Physically active (≥ 1.5 METs/day)	3038	4108	1070
Physically inactive (< 1.5 METs/day)	3804	4431	627
Alcohol consumption			
Never drinker	1340	1580	240
Light drinker	1306	1521	215
Moderate drinker	1466	1704	238
Heavy drinker	2591	3556	965
Number of health risk behaviours (current/former, heavy/light smoking, alcohol consumption (moderate or heavy), and physical inactivity)			
0	601	784	183
1	2468	3177	709
2	2674	3284	610
3	1101	1298	197
Health status			
Self-perceived general health			
Excellent	774	1242	468
Very good	2304	3106	803
Good	2595	2989	394
Fair	861	899	37.8
Poor	304	301	− 3.07
Self-perceived life stress			
Not at all stressful	809	874	65.1
Not very stressful	1478	1835	357
A bit stressful	2849	3642	793
Quite a bit stressful	1403	1817	414
Extremely stressful	283	351	67.9
Number of chronic conditions^‡^			
0	2182	3268	1086
1	1689	2174	485
2	1203	1337	134
3	768	777	9.18
4	427	419	− 7.51
5	226	216	− 9.85
6 or more	220	207	− 13.15

*Excluding Newfoundland and Labrador, Manitoba, British Columbia, and Yukon

^†^METs are metabolic equivalent of task (kcal/kg/day). For example, the “inactive” physical activity is equal to walking for exercise less than 30 min per day (3 METS/h)

^‡^Chronic conditions include self-reported asthma, arthritis, back problems, migraine headaches, chronic obstructive pulmonary disease, diabetes, hypertension, heart disease, cancer, intestinal ulcers, stroke, urinary incontinence, bowel disorder, mood disorder, and anxiety disorders

## Discussion

This study applied a validated risk prediction tool (OPoRT) to estimate the future 10-year burden of obesity in Canada using baseline risk factors attained through routinely collected surveillance data. Currently, there are risk prediction tools that have been developed for specific population subgroups. For example, a risk prediction model was developed for substantial weight gain, a risk factor for various metabolic diseases; however, this model is limited to middle-aged adults and is limited in accessibility in applied settings (Steffen et al. 2013). To the best of our knowledge, there are no population-based prediction models that estimate the burden of obesity that can be run using routinely collected risk factor information.

There are a number of frameworks in practice that provide a theoretical basis and an overarching structure for a set of strategies to tackle the burden of obesity. In the context of our results, a socio-environmental theoretical framework that considers the broader social determinants of obesity best aligns with the Ottawa Charter for Health Promotion (World Health Organization 1986). This theoretical model addresses physical, policy, economic and socio-cultural environments, in addition to targeting psychosocial and behavioural factors. Levering this theory aids in shifting our understanding of obesity from a focus on the influence of individual factors to a recognition of the more broad influences on health status that are now reflected in population increases in weight. In consideration of this framework, policies to modify elements in the daily environment in combination with actions from diverse sectors are likely to be more effective than policies that solely address personal responsibility (Roberto et al. 2015). Population-level strategies that focus on influencing both food choices (e.g., through clear nutrition labeling, leveraging price as an incentive, standards for advertising, reductions in fat, sugar and salt) and working with industry to improve the nutritional quality of foods (e.g., benchmarks to eliminate excess nutrients, reducing calorie or portion sizes, and standards in schools and workplaces) are among the most recommended strategies (World Cancer Research Fund 2016). In addition, policies aimed at improving the built environment and infrastructure, with the goal of promoting physical activity and improving the acceptability and safety of active transport, can also help reach many individuals in the population (Hallal et al. 2012). There is consensus, on the basis of research and practice, that public health strategies designed to tackle risk factors for obesity have been demonstrated to be cost-effective (Cecchini et al. 2010).

Another important consideration is the high risk and high burden of obesity predicted across age categories. Taking a life course perspective, the adolescent years can be a period where healthy or unhealthy habits are developed and often sustained into adulthood (Telama et al. 1997). Obesity is very difficult to treat once established (August et al. 2008), and it is important to consider that those who become obese at younger ages will experience a greater duration of time living with obesity, which has implications for long-term health. In addition to developing obesity early in life, evidence suggests that early adulthood is a common period of onset for overweight and obesity (Barbour-Tuck et al. 2018; Wisemandle et al. 2000) and transitioning from a childhood of normal weight status may convey equal or only slightly less risk (Juonala et al. 2011). From a preventive approach, interventions aimed at normal weight young adults may also be an important piece of a comprehensive strategy to reduce population obesity levels.

A number of studies have suggested that the association between income and obesity is sex-specific, although this pattern has been noted to differ between high-income and low-income countries (Dinsa et al. 2012). In high-income countries, the association between income and obesity is largely mixed for men and mainly negative for women (Dinsa et al. 2012). Consistent with previous studies (Public Health Agency of Canada and Canadian Institute for Health Information 2011), our results show an inverse relationship whereby as income increased, predicted burden of obesity decreased among women. However, among men, as income increased, predicted burden of obesity also increased. This inconsistency in association for men is also reinforced by results from the US National Health and Nutrition Examination Survey, which found no differences in obesity prevalence across income groups for men but an inverse pattern among women (Rubin 2018). These patterns emphasize the importance of sex considerations in obesity prevention policies.

Substantial interprovincial variations exist with a higher predicted burden of obesity in the Atlantic Provinces and in the Northwest Territories and Nunavut. The lowest predicted burden of obesity is expected to occur in British Columbia, Quebec, and Ontario. This trend may be explained, in part, by the large population of immigrants who live in these provinces and generally exhibit healthier behaviours than their Canadian-born counterparts (De Maio 2010; Lu et al. 2017). Mapping predicted regional rates of obesity across Canada over a decade should help researchers and public health decision markers identify where investments are especially needed to address obesity.

In 2013, the WHO developed a Global Non-communicable Disease (NCD) Action Plan, which outlines global targets for improving the prevalence of NCD risk factors (World Health Organization 2013). The framework includes obesity targets for adults and adolescents, calling for a zero increase in prevalence from 2010 to 2025. Despite a seemingly low bar, if trends in obesity persist in Canada, we are unlikely to meet this target; in fact, obesity prevalence is predicted to rise. Achievement of this target requires governments and health policy makers to take bolder, more definitive steps to increase obesity prevention across the population, not only among high-risk subgroups.

### Strengths and limitations

OPoRT has substantial applicability and is created to be accessible and transparent for use within applied settings, such as provincial ministries of health and regional health bodies. Specifically, these analyses support more precise and effective strategies for prevention that consider who is at risk and the burden at the population level. This model can be combined with more sophisticated microsimulation approaches to further quantify the health and economic impact of various strategies (Webber et al. 2014).

While predicting the future burden of obesity is useful for planning and resource allocation, our findings should be interpreted in light of certain limitations. First, OPoRT does not account for people who die during the time period, changes in population structure or migration. Those issues could be handled in a simulation framework, which can be integrated with OPoRT, something that has been done for other conditions (Manuel et al. 2014). Second, the CCHS sampling frame excludes approximately 2% of the population, including the institutionalized, individuals living on First Nations reserves, full-time residents of the Canadian Forces, and people living in certain remote regions. Given the data collection constraints on First Nations populations living on reserves in Canada, the design of the CCHS did not allow for inclusion of information on this important subgroup and as such, our results should be interpreted considering this limitation. Given that Indigenous people are at an increased risk of obesity, it is expected that our estimates may be underestimating the true obesity burden in Canada. Finally, given that the CCHS relies on self-reported data, there is potential for reporting bias, such as recall or social desirability bias. Reporting accuracy of participants affects all risks examined in this study. Estimates of burden are most affected when people report they are in the healthiest category (i.e., non-smoker or moderate drinker) when they are actually in a high-risk category, resulting in differential misclassification. However, to minimize this bias in our outcome, we applied a validated BMI correction equation, derived from the 2005 CCHS to all analyses, which have been shown to provide a more accurate estimate of an individual’s true weight status (Shields et al. 2011). Additionally, since BMI does not measure percent body fat directly and poorly distinguishes between total body fat and total body lean or bone mass, the use of BMI as an indicator of adiposity may not accurately reflect health risk for all individuals (De Lorenzo et al. 2013). In the future, a model that could predict abdominal obesity in the population, instead of only BMI, could enhance the model’s ability to inform obesity prevention in Canada.

This study provides a practical and meaningful way to better understand how the magnitude and distribution of obesity burden in the Canadian population can influence approaches to prevention. Most importantly, this research demonstrates a mechanism whereby routinely collected, publicly available data can be used to inform prevention and resource planning.

## Electronic supplementary material

ESM 1 (DOCX 48 kb)
