# Supplementary material for: The future burden of obesity in Canada: a modelling study
Source: Can J Public Health. 2019 Aug 19;110(6):768–78. doi: 10.17269/s41997-019-00251-y (PMC6900264; doi:10.17269/s41997-019-00251-y)
Supplement: Supplementary file 1 — (DOCX 48 kb) [file 41997_2019_251_MOESM1_ESM.docx]

**Supplementary File 1.** OPoRT function for males and females

| **Males** | | | **Females** | | |
| --- | --- | --- | --- | --- | --- |
| Predictors | Coefficients | P value | Predictors | Coefficients | P value |
| Intercept | 7.7187 | 0.386 | Intercept | -18.0238 | 0.0002 |
| Time | 0.5603 | <.0001 | Time | 0.5214 | <.0001 |
| Time^2^ | -0.0225 | 0.0002 | Time^2^ | -0.0169 | 0.0047 |
| BMI | -1.4073 | 0.0341 | BMI | 0.6156 | 0.101 |
| BMI^2^ | 0.0392 | 0.0015 | BMI^2^ | 0.0001 | 0.9884 |
| Age | -0.0208 | <.0001 | Age | -0.0223 | <.0001 |
| Age X Time | -0.005 | <.0001 | Age X Time | -0.0038 | <.0001 |
| Obese X BMI | 0.6968 | <.0001 | Obese X BMI | 0.2808 | 0.0061 |
| Obese X BMI^2^ | -0.0234 | <.0001 | Obese X BMI^2^ | -0.0093 | 0.0059 |
| Obese X Age | 0.0203 | 0.0069 | Obese X Age | 0.0216 | 0.0019 |
| Obese X Age X Time | -0.0043 | <.0001 | Obese X Age X Time | -0.0048 | <.0001 |
| Former Smoker | 0.2979 | 0.0024 | Former Smoker | 0.0538 | 0.5668 |
| Current Smoker | 0.3182 | 0.0024 | Current Smoker | 0.2463 | 0.028 |
| Live with a spouse/partner | -0.2332 | 0.0714 | Live with a spouse/partner | -0.1879 | 0.115 |
| Parent living with a spouse/partner and children | -0.1758 | 0.1433 | Parent living with a spouse/partner and children | -0.2145 | 0.0969 |
| Single parent living with children | 0.4612 | 0.1628 | Single parent living with children | -0.2659 | 0.1291 |
| Other living arrangement | -0.0497 | 0.7483 | Other living arrangement | 0.0885 | 0.5789 |
| Any post-secondary education | -0.1472 | 0.0877 | Physically inactive | 0.233 | 0.0061 |
| Non-drinker | 0.2336 | 0.0591 | Asian | -1.2186 | 0.0096 |
|  |  |  | Aboriginal | 0.5767 | 0.3065 |
|  |  |  | South Asian | -0.4955 | 0.0901 |
|  |  |  | Other | 0.4868 | 0.2198 |
|  |  |  | Black | 0.2534 | 0.343 |
| Reference groups: never smoker, unattached individual living alone, white ethnicity | | | | | |

Reference: Lebenbaum M, Espin-Garcia O, Li Y, Rosella LC (2018) Development and validation of a population based risk algorithm for obesity: The Obesity Population Risk Tool (OPoRT). PLOS ONE 13(1): e0191169. <https://doi.org/10.1371/journal.pone.0191169>.

**Supplementary File 2.** Definitions for descriptive variables

| **Risk Factor** | **CCHS survey question(s) used to define descriptive variables** |
| --- | --- |
| Smoking Status | Type of smoker  How many cigarettes do you smoke each day now? (daily smokers)  On days that you smoke, how many cigarettes do you usually smoke? (occasional smokers)  How many cigarettes did you usually smoke each day? (former daily smokers)  In your lifetime, have you smoked a total of 100 or more cigarettes (about 4 packs)? |
| Physical Activity | Daily energy expenditure in leisure time physical activities in the past 3 months expressed as Metabolic Equivalent of Task (MET) (kcal/kg/day).  Energy expenditure calculated using the frequency and duration per session of physical activity and MET value of the activity. |
| Alcohol Consumption | During the past 12 months, have you had a drink of beer, wine, liquor or any other alcoholic beverage?  During the past 12 months, how often did you drink alcoholic beverages?  How often in the past 12 months have you had 5 or more drinks on one occasion?  Number of drinks – past week  Starting with yesterday, that is [day name], how many drinks did you have – Monday  Starting with yesterday, that is [day name], how many drinks did you have – Tuesday  Starting with yesterday, that is [day name], how many drinks did you have – Wednesday  Starting with yesterday, that is [day name], how many drinks did you have – Thursday  Starting with yesterday, that is [day name], how many drinks did you have – Friday  Starting with yesterday, that is [day name], how many drinks did you have – Saturday  Starting with yesterday, that is [day name], how many drinks did you have - Sunday |
| Number of chronic conditions | Asthma- Have you had asthma symptoms or asthma attacks in the past 12 months?  Arthritis- Do you have arthritis, excluding fibromyalgia?  Back problems- Do you have back problems, excluding fibromyalgia and arthritis?  Migraines- Do you have migraine headaches?  Chronic obstructive pulmonary disease- Do you have bronchitis, emphysema or chronic obstructive pulmonary disease?  Diabetes- Do you have diabetes?  High blood pressure- Do you have high blood pressure?  Heart disease- Do you have heart disease?  Cancer- Do you have cancer?  Intestinal ulcers- Do you have intestinal or stomach ulcers?  Stroke- Do you suffer from the effects of stroke?  Urinary incontinence- Do you have urinary incontinence?  Bowel disease- Do you have a bowel disorder such as Crohn’s Disease, ulcerative colitis, irritable Bowel syndrome or bowl incontinence?  Mood disorder- Do you have a mood disorder such as depression, bipolar disorder, mania or dysthymia?  Anxiety disorder- Do you have an anxiety disorder such as a phobia, obsessive-compulsive disorder or panic disorder? |

**Supplementary File 3**. Sensitivity Analysis

**Table 2** 10-year predicted obesity burden among adult Canadians by socio-economic, behavioural and health status characteristics (2013/14-2023/24)

| Characteristic | Predicted burden of obesity by population sub group (cases per 1,000)(95% CI) | Total predicted number of cases with obesity (thousands) |
| --- | --- | --- |
| Total predicted number of obese cases | 326 (322, 329) | 8,530 |
| Socio-economics | | |
| Sex |  |  |
| Male | 347 (341, 354) | 4,564 |
| Female | 304 (299, 309) | 3,966 |
| Age group |  |  |
| <35 | 329 (322, 337) | 2,443 |
| 35-49 | 373 (364, 382) | 2,526 |
| 50-64 | 337 (330, 344) | 2,439 |
| 65+ | 236 (231, 241) | 1,122 |
| Ethnicity |  |  |
| White | 338 (334, 342) | 6,617 |
| Visible minority | 287 (277, 297) | 1,670 |
| Immigrant status |  |  |
| Canadian-born | 349 (345, 353) | 6,610 |
| Immigrant | 261 (252, 270) | 1,674 |
| Food security^*^ |  |  |
| Food secure | 323 (319, 328) | 6,206 |
| Moderately food insecure | 409 (388, 429) | 407 |
| Severely food insecure | 454 (417, 491) | 216 |
| Behavioural | | |
| Smoking status |  |  |
| Heavy smoker (1+ packs/day) | 393 (369, 417) | 283 |
| Light smoker (< 1 pack/day) | 343 (333, 353) | 1,464 |
| Former (heavy) smoker | 415 (403, 428) | 661 |
| Former (light) smoker | 337 (328, 345) | 1,545 |
| Non-smoker | 304 (298, 310) | 4,273 |
| Physical activity^†^ |  |  |
| Physically active (≥1.5 METs/day) | 292 (287, 296) | 4,086 |
| Physically inactive (<1.5 METs/day) | 365 (359, 371) | 4,445 |
| Alcohol consumption |  |  |
| Never drinker | 321 (312, 330) | 1,582 |
| Light drinker | 365 (354, 375) | 1,523 |
| Moderate drinker | 273 (266, 279) | 1,694 |
| Heavy drinker | 345 (339, 351) | 3,556 |
| Number of health risk behaviours  (current/ former, heavy/light smoking, alcohol consumption (moderate or heavy), and physical inactivity) | | |
| 0 | 277 (266, 288) | 776 |
| 1 | 304 (298, 310) | 3,170 |
| 2 | 346 (339, 352) | 3,279 |
| 3 | 374 (363, 385) | 1,305 |
| Health status | | |
| BMI Category |  |  |
| Overweight (BMI:25.0-29.9) | 285 (281, 289) | 2,650 |
| Self-perceived general health |  |  |
| Excellent | 226 (219, 233) | 1,222 |
| Very good | 308 (302, 314) | 3,095 |
| Good | 386 (379, 393) | 2,996 |
| Fair | 408 (395, 420) | 906 |
| Poor | 414 (386, 442) | 304 |
| Self-perceived life stress |  |  |
| Not at all stressful | 294 (283, 304) | 870 |
| Not very stressful | 305 (299, 312) | 1,825 |
| A bit stressful | 327 (321, 333) | 3,638 |
| Quite a bit stressful | 353 (344, 362) | 1,822 |
| Extremely stressful | 387 (365, 409) | 351 |
| Number of chronic conditions^‡^ |  |  |
| 0 | 287 (281, 294) | 3,259 |
| 1 | 322 (315, 330) | 2,162 |
| 2 | 347 (338, 357) | 1,333 |
| 3 | 386 (374, 398) | 777 |
| 4 | 418 (401, 435) | 422 |
| 5 | 451 (428, 474) | 219 |
| 6 or more | 507 (478, 535) | 211 |

^*^Excluding Newfoundland and Labrador, Manitoba, British Columbia, and Yukon.

^†^METs are Metabolic Equivalent of Task (kcal/kg/day). For example, the “inactive” physical activity is equal to walking for exercise less than 30 min per day (3 METS/hr).

^‡^Chronic conditions include self-reported asthma, arthritis, back problems, migraine headaches, chronic obstructive pulmonary disease, diabetes, hypertension, heart disease, cancer, intestinal ulcers, stroke, urinary incontinence, bowel disorder, mood disorder, and anxiety disorders.

**Table 3** 10-year predicted change in burden of obesity among adult Canadians by socio-economic, behavioural and health status characteristics (2013/14-2023/24)

| Characteristic | Total baseline number of cases with obesity (thousands) | Total predicted number of cases with obesity (thousands) | Incident number of cases with obesity (thousands) |
| --- | --- | --- | --- |
| Total predicted number of obese cases | 6,844 | 8,530 | 1,686 |
| Socio-economics | | | |
| Sex | | | |
| Male | 3,663 | 4,564 | 901 |
| Female | 3,181 | 3,966 | 785 |
| Age group | | | |
| <35 | 1,399 | 2,443 | 1,044 |
| 35-49 | 1,889 | 2,526 | 637 |
| 50-64 | 2,269 | 2,439 | 170 |
| 65+ | 1,288 | 1,122 | -166 |
| Ethnicity | | | |
| White | 5,517 | 6,617 | 1,101 |
| Visible minority | 1,129 | 1,670 | 541 |
| Immigrant status | | | |
| Canadian-born | 5,374 | 6,610 | 1,236 |
| Immigrant | 1,271 | 1,674 | 403 |
| Food security^*^ | | | |
| Food secure | 4,981 | 6,206 | 1,224 |
| Moderately food insecure | 320 | 407 | 86.3 |
| Severely food insecure | 175 | 216 | 41.2 |
| Behavioural | | | |
| Smoking status | | | |
| Heavy smoker (1+ packs/day) | 231 | 283 | 51.9 |
| Light smoker (< 1 pack/day) | 1,030 | 1,464 | 434 |
| Former (heavy) smoker | 667,561 | 661 | -68.8 |
| Former (light) smoker | 1,328 | 1,545 | 216 |
| Non-smoker | 3,336 | 4,273 | 937 |
| Physical activity^†^ | | | |
| Physically active (≥1.5 METs/day) | 3,038 | 4,086 | 1,048 |
| Physically inactive (<1.5 METs/day) | 3,806 | 4,445 | 638 |
| Alcohol consumption | | | |
| Never drinker | 1,340 | 1,582 | 242 |
| Light drinker | 1,306 | 1,523 | 218 |
| Moderate drinker | 1,466 | 1,694 | 228 |
| Heavy drinker | 2,591 | 3,556 | 964 |
| Number of health risk behaviours  (current/ former, heavy/light smoking, alcohol consumption (moderate or heavy), and physical inactivity) | | | |
| 0 | 601 | 776 | 175 |
| 1 | 2,468 | 3,170 | 702 |
| 2 | 2,674 | 3,279 | 605 |
| 3 | 1,100 | 1,305 | 204 |
| Health Status | | | |
| Self-perceived general health | | | |
| Excellent | 774 | 1,222 | 448 |
| Very good | 2,304 | 3,095 | 792 |
| Good | 2,595 | 2,996 | 401 |
| Fair | 861 | 906 | 44.7 |
| Poor | 304 | 304 | 5.42 |
| Self-perceived life stress | | | |
| Not at all stressful | 809 | 870 | 61.1 |
| Not very stressful | 1,478 | 1,825 | 348 |
| A bit stressful | 2,849 | 3,638 | 789 |
| Quite a bit stressful | 1,403 | 1,822 | 419 |
| Extremely stressful | 283 | 351 | 67.7 |
| Number of chronic conditions^‡^ | | | |
| 0 | 2,182 | 3,259 | 1,078 |
| 1 | 1,689 | 2,162 | 473 |
| 2 | 1,203 | 1,333 | 130 |
| 3 | 768 | 777 | 9.25 |
| 4 | 427 | 422 | -4.93 |
| 5 | 226 | 219 | -7.47 |
| 6 or more | 221 | 211 | -9.55 |

^*^Excluding Newfoundland and Labrador, Manitoba, British Columbia, and Yukon.

^†^METs are Metabolic Equivalent of Task (kcal/kg/day). For example, the “inactive” physical activity is equal to walking for exercise less than 30 min per day (3 METS/hr).

^‡^Chronic conditions include self-reported asthma, arthritis, back problems, migraine headaches, chronic obstructive pulmonary disease, diabetes, hypertension, heart disease, cancer, intestinal ulcers, stroke, urinary incontinence, bowel disorder, mood disorder, and anxiety disorders.
